# *Crocodylus porosus* Gut Bacteria: A Possible Source of Novel Metabolites

**DOI:** 10.3390/molecules26164999

**Published:** 2021-08-18

**Authors:** Naveed Ahmed Khan, Morhanavallee Soopramanien, Sutherland Kester Maciver, Tengku Shahrul Anuar, Kuppusamy Sagathevan, Ruqaiyyah Siddiqui

**Affiliations:** 1 Department of Clinical Sciences, College of Medicine, University of Sharjah, Sharjah 26666, United Arab Emirates; sagatk@sunway.edu.my; 2 Department of Biological Sciences, Sunway University, Petaling Jaya 47500, Selangor, Malaysia; morhana07@gmail.com; 3 Centre for Discovery Brain Science, Edinburgh Medical School, Biomedical Sciences, University of Edinburgh, Edinburgh EH8 9YL, UK; smaciver@ed.ac.uk; 4 Centre of Medical Laboratory Technology, Faculty of Health Sciences, Puncak Alam Campus, Universiti Teknologi MARA, Kuala Selangor 42300, Selangor, Malaysia; tengku9235@uitm.edu.my; 5 College of Arts and Sciences, American University of Sharjah, Sharjah 26666, United Arab Emirates; rsiddiqui@aus.edu

**Keywords:** crocodilians, crocodiles, microbiome, *Crocodylus porosus*, gut microbiota, novel metabolites

## Abstract

Crocodiles are remarkable animals that have the ability to endure extremely harsh conditions and can survive up to a 100 years while being exposed to noxious agents that are detrimental to *Homo sapiens*. Besides their immunity, we postulate that the microbial gut flora of crocodiles may produce substances with protective effects. In this study, we isolated and characterized selected bacteria colonizing the gastrointestinal tract of *Crocodylus* *porosus* and demonstrated their inhibitory effects against three different cancerous cell lineages. Using liquid chromatography-mass spectrometry, several molecules were identified. For the first time, we report partial analyses of crocodile’s gut bacterial molecules.

## 1. Introduction

Crocodilians and birds are the only extant archosaurs, having survived the catastrophic Cretaceous–Tertiary extinction event, while other members of this clade such as dinosaurs and pterosaurs have become extinct [1]. Of note, *Homo sapiens* is just one species amid millions of others and we are a moderately new addition to this planet. Lessons may be learned from species such as crocodiles that have successfully adapted and evolved over millions of years. Crocodilians in sanctuaries and farms are normally exposed to radiation, heavy metals, diet of rotten meat, pollutants, etc. and have a prolonged lifespan of up to 100 years [2,3,4,5,6]. Even with exposure to stressful environments and carcinogenic materials, these species thrive under conditions that are considered unfavorable to *Homo sapiens*. The basis of their endurance is not clear and thus we speculated that such species may have mechanisms to protect themselves from noxious agents and to endure such longevity [7]. Previously, we have suggested that the ability of animals such as crocodiles that reside in polluted habitats and thrive in pathogen abundant environments could be due to either a highly adapted immune system and/or their gut microbiota may contribute to their resilience [8,9]. In this study, we hypothesized that selected bacteria isolated from crocodile gut exhibit properties against cancer cell lines. Among many other possible sources, we investigated the effect of metabolites from such as under-investigated source (i.e., crocodile gut).

In support of the latter, the gut microbiome is known to perform key roles in regulating the behavior and health of its host [10]. There has been an increase in research on studies of the human microbiome. Various work has depicted that the microbiome is known to provide protection against a variety of disorders such as systemic metabolic disease (type 2 diabetes and obesity), inflammatory bowel disease, allergic reactions, and atopic eczema [11,12,13,14]. Furthermore, research has disclosed that the microbiome may offer protection against neurodevelopment disorders like schizophrenia and autism spectrum disorders (ASD) [11,12], whereas it has also been reported that the gut microbiota can have beneficial properties against cancer [13,14,15]. However, there have been few studies on the gut microbiome of reptiles, and crocodiles in particular [16]. In a recent study, it was shown that microbiome composition of alligator is dominated by phyla represented by *Firmicutes*, mainly pathogenic *Clostridia* and *Fusobacteria*, which is different from mammals, fish, other reptiles, which are mainly dominated by *Firmicutes* and *Bacteroidetes* [17]. It is interesting to note an abundance of pathogenic bacteria in alligator’s microbiome that may play a role in host physiology. The goal of this study was to isolate and characterize selected gut bacteria of the saltwater crocodile, *Crocodylus porosus*. Conditioned media from the selected gut bacteria were assessed for potential activity against three cancerous cell lines through cell metabolic activity via the MTT 3-(4,5-dimethylthiazol-2-yl)-2,5-diphenyltetrazolium bromide assays and cell survival assays. Subsequently, we conducted liquid chromatography-mass spectrometry (LC-MS) to determine the molecules present in active conditioned media. This is the first to explore the presence of potential molecules from the selected gut bacteria of *C. porosus*.

## 2. Results

### 2.1. Several Gut Bacteria Were Isolated from Crocodylus porosus

Several bacteria were isolated from the various sections of the gastrointestinal tract of *C. porosus*; mouth and esophagus, stomach, small intestine, large intestine, and anus. These bacteria are detailed in Table 1. In this study, we identified *Proteobacteria*, *Firmicutes*, *Actinobacteria*, *Bacteroidetes*, and *Deinococcus*-*Thermus*.

### 2.2. Selected Conditioned Media from Bacteria Isolated from C. porosus Affected Cell Viability

*P. guezennei* isolated from the duodenum, *P. aeruginosa* isolated from the small intestine, and *A. dhakensis* isolated from the large intestine of *C. porosus* inhibited the cell metabolic ability of the selected cancer cell lines (HeLa, MCF-7 and PC3) as well as HaCat cells (Table 2) significantly (*p* < 0.05 using two sample *t*-test, two-tailed distribution), albeit activity was reduced against HaCat cells. Moreover, *P. aeruginosa* from stomach, *O. oryzae* from duodenum, *P. aeruginosa* from large intestine, and *P. rettgeri* from anus, reduced the viability of MCF-7, PC3 cells by more than 40% (Table 2). Furthermore, *P. hauseri*, *H. paralvei,* and *S. pasteuri* from stomach, *K. pneumoniae* from duodenum, and *P. vulgaris* and *P. rettgeri* from small intestines reduced the metabolic activity of MCF-7 cells by more than 40%. While *P. hauseri* from small intestine and *P. columbae* from the anus affected the viability of MCF-7 cells only (Table 2). Several conditioned media from bacteria isolated from the gastrointestinal tract of *C. porosus* did not affect the cell metabolic activities of the cell lines tested (data not shown). However, conditioned media exhibiting prominent effects are shown in Table 2. The remaining bacteria showed minimal effects (less than 10% inhibition) (data shown in Table S1).

**Figure 1:**
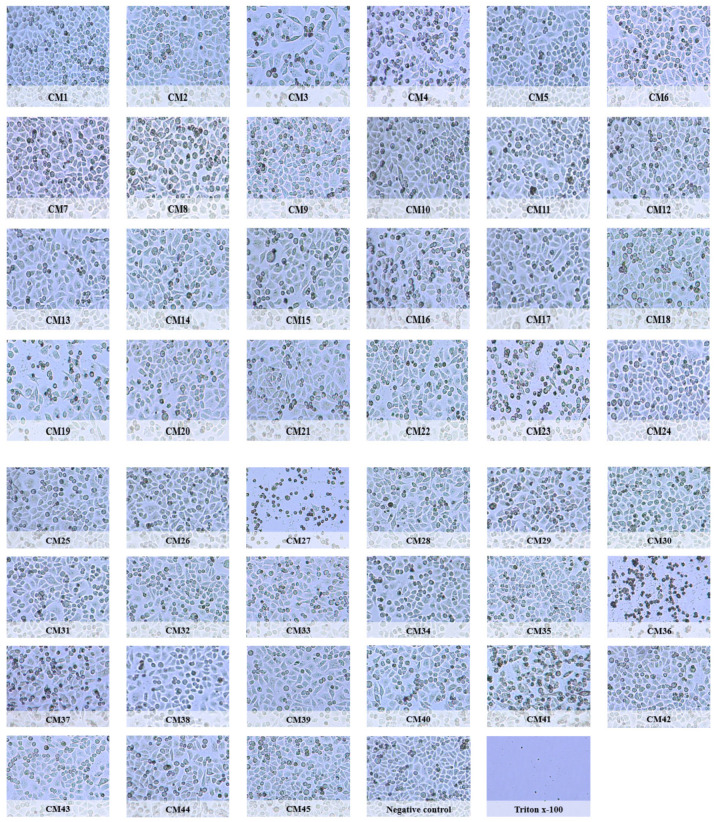
Representative images of cell survival assay results for HeLa cells treated with *C. porosus* conditioned media. HeLa cells were treated with 100 µL conditioned media from *C. porosus* for 24 h at 37 °C in carbon dioxide. Positive control cells were treated with Triton X-100. The cells were trypsinized and seeded into a new plate. Microscopic images were taken after 24 h using an inverted light microscope at ×40 magnification. [(CM1: *D. grandis*, CM2: *O. intermedium*, CM3: *P. aeruginosa*, CM4: *B. sediminis*, CM5: *M. paraoxydans*, CM6: *E. tarda*, CM7: *A. hermannii*, CM8: *T. spumae*, CM9: *D. polyhydroxybutyrativorans*, CM10: *S. pasteuri*, CM11: *E. cloacae*, CM12: *C. yeoncheonense*, CM13: *B. aryabhattai*, CM14: *S. aureus*) from mouth and esophagus, (CM15: *P. hauseri*, CM16: *H. paralvei*, CM17: *S. pasteuri*, CM18: *A. salmonicida*, CM19: *P. aeruginosa*) from stomach, (CM20: *K. quasipneumoniae*, CM21: *S. pasteuri*, CM22: *K. pneumoniae*, CM23: *P. guezennei*, CM24: *O. oryzae*, CM25: *E. cloacae*) from duodenum, (CM26: *P. vulgaris*, CM27: *P. aeruginosa*, CM28: *P. vulgaris*, CM29: *P. hauseri*, CM30: *P. alcalifaciens*, CM31: *P. rettgeri*) from small intestine, (CM32: *P. mirabilis*, CM33: *P. hauseri*, CM34: *P. alcalifaciens*, CM35: *P. shigelloides*, CM36: *A. dhakensis*, CM37: *P. aeruginosa*, CM38: *P. vulgaris*) from large intestine, and (CM39: *P. columbae*, CM40: *P. terrae*, CM42: *A. dhakensis*, CM43: *P. rettgeri*, CM44: *P. mirabilis*, CM45: *P. mirabilis*) from anus.

Representative effects of selected CM were observed using cell survival assays. *P. guezennei* (CM23) isolated from the duodenum, *P. aeruginosa* (CM 27) isolated from the small intestine, and *A. dhakensis* (CM36) isolated from the large intestine of *C. porosus* inhibited the survival of HeLa cells. Post-treatment, cells treated with CM23, CM27, and CM36 appeared round, while the remaining conditioned media did not affect the survival of HeLa cells as the cell morphology was similar to the negative control (Figure 1).

### 2.3. Selected Conditioned Media from C. porosus Possess Molecules with Anticancer Activity

Active CM of *P. guezennei* (duodenum), *P. aeruginosa* (small intestine), and *A. dhakensis* (large intestine) were subjected to LC-MS. Spectra for negative and positive polarity detected 60 molecules from *P. guezennei* CM. All molecules identified using LC-MS are shown in Table S2. Out of 60 molecules, 31 metabolites were identified (Table S2A), while the remaining 29 molecules remained unidentified (Table S2B). For the unidentified molecules, mainly the retention time and molecular mass were identified and, in some cases, the molecular formula was also obtained. One of the molecule with reported anticancer activity was l,l-Cyclo(leucylprolyl). Moreover, some of the identified molecules had various reported activities such as antibacterial activity (lactic acid, f-Honaucin A, and l,l-Cyclo(leucylprolyl), antifungal activity (3-hydroxy-decanoic acid), etc. For *P. aeruginosa* CM, 82 molecules were identified for both negative and positive ion polarity. Out of 82 molecules, 26 were identified (Table S3A), some of which had anticancer and antimicrobial properties (Table S3). For *A. dhakensis*, a total of 20 molecules were detected through LC-MS, out of which seven were identified (Table S4A) while 13 remained unidentified (Table S3B).

## 3. Discussion

Among many other possible sources, we investigated the effect of metabolites from an under-investigated source such as crocodile gut. The overall aim of the study was to determine whether the selected gut bacteria of crocodile may be a repertoire of potential novel molecules. Selected bacteria inhabiting various regions of the gastrointestinal tract were isolated and identified. Most prior studies conducted on the microbiota of crocodile involved characterization of bacteria isolated from cloacal swabs [18,19]. Using 16S rDNA sequencing, the results showed 30 diverse bacterial species including *Proteobacteria*, *Firmicutes*, *Actinobacteria*, *Bacteroidetes*, and *Deinococcus-Thermus*. These findings are supported by previous studies whereby it was determined that the gut microbiota of lizards was mostly colonized by *Firmicutes* (33.2–73%), *Bacteroidetes* (6.2–45.7%), and *Proteobacteria* (5.7–62.3%) [19]. One of the limitations of this study was that we focused on aerobic culturable bacteria and future studies are needed to determine anaerobic bacteria and other unculturable bacteria/other microbes that may also be a potential source of novel molecules shielding the crocodile from adverse conditions. Consequently, these microorganisms should also be recovered and the activities of their metabolites assessed and is the subject of future studies.

The conditioned media from the crocodile gut bacteria were prepared and their effects on various cell lines were determined. Primary metabolites are secreted by bacteria to promote their growth, maturation, and replication, while secondary metabolites are usually secreted when bacteria are under stress. Moreover, secondary metabolites have various properties such as antibiotic effects, enzyme inhibitors, immunomodulators, antitumor agents, and growth promoters of cells [20]. Notably, we used in vitro assays in the present study to determine the effects of the CM against cancer cell lines. Although our findings cannot be applied directly into clinical practice, it is an acceptable approach at an early stage of research in drug discovery from a potentially interesting source. Further research to validate these findings in vivo will determine the translational value of these findings.

Of note, three bacterial species isolated from the gastrointestinal tract of *C. porosus* exhibited inhibition of cell metabolic activity or viability reduction, and cell survival inhibition. These included conditioned media prepared from *P. guezennei* from the duodenum, *P. aeruginosa* from the small intestine, and *A. dhakensis* from the large intestine of *C. porosus*. Previous studies have reported that *P. aeruginosa* synthesize and secrete azurin, a bacterial peptide that exhibits anticancer activity by promoting apoptosis in cells [21,22]. Of note, *P. aeruginosa* isolated from different locations of the gut inhibited growth of all cancer cells tested, regardless of the site of isolation, albeit some variations were observed, as indicated in Table 2. However, some variations are expected and are likely due to different growth conditions in different parts of the GI tract and/or experimental conditions of the bacterial CM preparations and/or the MTT assay. For example, *P. aeruginosa* isolated from the mouth showed inhibition of 89%, 64%, and 67% against HeLA, MCF-7, and PC3, respectively; *P. aeruginosa* isolated from stomach showed inhibition of 66%, 51%, and 53% against HeLA, MCF-7, and PC3, respectively; *P. aeruginosa* isolated from small intestine showed inhibition of 52%, 25%, and 40% against HeLA, MCF-7, and PC3, respectively; and *P. aeruginosa* isolated from the large intestine showed inhibition of 66%, 56%, and 46% against HeLA, MCF-7, and PC3, respectively. These findings showed that CM from *P. aeruginosa* exhibited significant inhibition against all cell lines tested (*p* < 0.05), however there is variability between them depending on their origin. This is the first time *P. aeruginosa* has been isolated from crocodile gut and grown in culture and this variability could be attributed to the source, the isolation site, the environmental conditions, or other factors such as CM preparations, and it is the subject of future studies. Overall, CM of *P. aeruginosa* exhibited inhibition of all cancer cell lines tested, which clearly demonstrate inhibitory effects. Notably, the selected CM showed reduced activity against normal HaCat cells. For example, CM from *P. aeruginosa* inhibited cells by 89.5% against HeLa cells, versus 59.0% against normal HaCat cells. Despite activity against normal cell lines, it is suggested that any potentially active molecule(s) can be used in the rational development of targeted chemotherapeutic approaches through conjugation with antigen-specific treatment and/or localized delivery.

Following LC-MS analysis, 60, 82, and 20 molecules from conditioned media prepared from *P. guezennei*, *P. aeruginosa*, and *A. dhakensis*, respectively, were detected. Although precise fragmentation is needed to validate the identity of these molecules, and this is a limitation of the present study, however, best matches were identified from the respective conditioned media from the Metlin library database. LC-MS results revealed that conditioned media prepared from *P. guezennei* isolated from the duodenum of *C. porosus* possessed a molecule with previously reported anticancer activity, namely; l,l-Cyclo(leucylprolyl). l,l-cyclo(leucylprolyl) is a flavonoid that exhibits anticancer effects against human cervical carcinoma cells and Glioma UG-87 cells using the MTT assay [23]. Similar results were obtained from the LC-MS analysis of conditioned media of *P. aeruginosa* and *A. dhakensis*. For example, various molecules were identified including antibacterial activity (lactic acid [24], f-Honaucin A [25], l,l-Cyclo(leucylprolyl) [26], antifungal activity [3-hydroxy-decanoic acid [27]), however, the properties and activity of the majority of molecules remain unidentified and are the subject of future studies.

The unidentified molecules may be novel. Although these findings are acquired using well-known METLIN Metabolomics Database (i.e., a repository for mass spectrometry metabolomics data), which is designed to aid in metabolite identification using this feature-rich, comprehensive metabolite, and tandem mass spectrometry database designed for untargeted metabolomic analysis, these findings should be interpreted with caution. Future studies are needed to validate these findings. Of note, as bacteria were cultured in RPMI-1640, the composition of RPMI-1640 includes aspartic acid; glutamic acid; asparagine; serine; glutamine; histidine; glycine; threonine; arginine; alanine; tyrosine; cystine; valine; methionine; norvaline; tryptophan; phenylalanine; isoleucine; leucine; lysine; hydroxyproline; sarcosine; proline; aminobenzoic acid; choline chloride; folic acid; inositol; picotinamide; pantothenic acid; hemicalcium; pyridoxine; riboflavin; thiamine; vitamin B12; glucose; glutathione; and sodium bicarbonate. As some of these compounds such as l-glutamate were also identified in bacterial CM identified using LC-MS (Supplementary Tables), their source should be interpreted with caution.

Overall, the identity and potential effects of these molecules alone or in combination needs further validation and will be the subject of future studies. For example, it is unclear whether the metabolites produced by crocodile gut bacteria in vivo and in vitro are the same. This would need to be determined through direct LC-MS analysis of gut content of crocodile as well as bacterial CM and is the subject of future studies. Furthermore, there is a need to identify uncultured bacteria and anaerobes and possibly other microbial presence to fully understand the total gut microbiome of the crocodile gut, in addition to determining the effects of each molecule on different cancer cells, in vitro and in vivo as well as determine their molecular mechanisms of action. The present study involving sample collection from a crocodile and data obtained from subsequent experiments conducted, are hypothesis-confirming experiments and further molecular studies are needed to validate these findings and determine their potential translational value using a large sample size.

For the first time, such a study was accomplished whereby the culturable gut microbiota of these reptiles were isolated and identified and their various effects were determined. Herein, we presented that the selected gut bacteria of *C. porosus* displayed potent activities, and have elucidated various molecules that could serve as possible drug leads; however, further research is needed to achieve these anticipations.

## 4. Methods and Materials

### 4.1. Ethics Committee Approval and Procurement of Crocodile

The Department of Wildlife and National Parks (PERHILITAN), Malaysia, permitted the use of crocodile material. Moreover, Sunway Research Ethics Committee, Sunway University, Malaysia approved the study (Research Ethics Approval Code: SUNREC 2019/023). A convention on international trade in endangered species (CITES) of wild fauna and flora registered crocodile farm, provided the saltwater crocodile, *Crocodylus porosus*. Management of crocodile including anesthesia and dissection of the internal organs were carried out by qualified personnel at the farm who routinely perform these procedures.

The crocodile used in this study was four years old, 205 cm in length, and 46 cm wide with a weight of 28.2 kg, belonging to the species, *Crocodylus porosus*. The specimen was active and in good health with no apparent symptom of sickness and disease. The gastrointestinal tract measured 115 cm long in situ and after uncoiling, the intestines comprised of the large and small intestines, measuring 210 cm in length. The gastrointestinal tract from the esophagus to the anus measured 267 cm in length. Longitudinal incisions were then made along different sections of the gastrointestinal tract of the crocodile including the esophagus, gall bladder, stomach, large and small intestines, pancreas, and anus. Using sterile cotton swabs, the microbiota inhabiting those sections of the gastrointestinal tract were isolated and inoculated onto nutrient (CM0003B, Oxoid Limited, Basingstoke, UK) and blood agar plates (CM0854, Oxoid, UK) and kept overnight at 37 °C.

### 4.2. Bacterial Identification

After incubation, several colonies of bacteria were observed on nutrient and blood agar plates. These colonies were separated according to shape, color, texture, and size, and individual colonies were selected and inoculated onto fresh agar plates and incubated overnight at 37 °C. In this way, pure cultures were obtained on individual agar plates. Bacteria were identified through 16S ribosomal DNA sequencing.

### 4.3. 16S rDNA Sequencing

A single bacterial colony was obtained and inoculated into fresh nutrient broth (CM0001, Oxoid, Basingstoke, UK) and was kept overnight at 37 °C in an incubator shaker. The bacterial suspension was then centrifuged at 700× *g* for 15 min at 37 °C, and the bacterial nucleic acids were extracted for sequencing using the QIAGEN DNA Extraction Kit, as detailed in instructions provided by the manufacturer (Cat. No. 47054, Qiagen, Germantown, MD, USA). The extracted DNA was then amplified using Taq DNA Polymerase 2X-preMix (Qiagen) and a pair of 16S rDNA Universal primers: 27F (5′-AGAGTTTGATCCTGGCTCAG-3′) forward primer and 1492R (5′-GGTTACCTTGTTACGACTT-3′) reverse primer. Polymerase chain reaction (PCR) was accomplished with the following conditions: initial denaturation step; one cycle at 95 °C for 5 min, amplification step; 30 cycles (i) 95 °C for 30 s, (ii) 55 °C for 30 s, and (iii) 72 °C for 1 min and final step; one cycle at 72 °C for 5 min. To ensure that the 16S DNA of the bacteria was amplified, gel electrophoresis of the PCR product was accomplished using 1.5% agarose gel (Sigma-Aldrich, St. Louis, MO, USA) in 1× Tris-acetate-EDTA (TAE) buffer at 100 V for 40 min alongside DNA loading dye and a 1 kb DNA ladder (Srinivasan et al., 2015). The PCR amplified product was sequenced commercially by the Sanger sequencing method to obtain the nucleotide sequence [8,28]. The alignment of nucleotide sequences was achieved via ChromasPro software and the resulting sequences were exported into alignment tool “Basic Local Alignment Search Tool” (BLAST) to identify matches with existing reference sequences. The sequences with the highest and lowest values calculated were then determined more precisely using pairwise BLASTN. The results were reflected as valid if the homologue rate was greater than 99% [28].

### 4.4. Preparation of Conditioned Media

Conditioned media (CM) is a suspension of the primary and secondary bacterial metabolites. Each selected single bacterial colony was suspended in Rosewell Park Memorial Institute (RPMI-1640) medium (Invitrogen, Waltham, MA, USA) and kept for 48 h at 37 °C. The bacterial suspension was then centrifuged at 4 °C, 6000× *g*, for 1 h. The supernatant containing the bacterial metabolites was sterile filtered with a cellulose acetate syringe filter (cat. no. 17573, Sartorius, Göttingen, Germany) of 0.22 μm pore size (to ensure that CM was bacteria free) and was stored at −80 °C until required.

### 4.5. Cell Cultivation

To evaluate the potential effects of the CM, three cancer cell lines were maintained. The cancer cells used in this study were breast cancer (MCF7 (ATCC^®^ HTB-22™)), cervical cancer (HeLa (ATCC^®^ CCL2™)), and prostate cancer (PC3 (ATCC^®^ CRL1435™)). These cells were acquired from the American Type Culture Collection. The aforementioned cell lines were selected as they represent different types of cancers in different organ systems. In addition, a normal cell line, aneuploid immortal keratinocyte (HaCaT) (CLS 300493, CLS Germany, Hamburg, Germany), was used to determine the CM effects. All the cells were cultivated in RPMI-1640 complemented with 10% fetal bovine serum (FBS), 1% Minimum Essential Media Non-Essential amino acid (MEM NEAA), 1% l-glutamine, and 1% penicillin streptomycin antibiotic (Invitrogen) at 37 °C, with a supply of 5% carbon dioxide and 95% humidity, as detailed previously [29,30,31].

### 4.6. 3-(4,5-Dimethylthiazol-2-yl)-2,5-diphenyltetrazolium Bromide (MTT) Cell Metabolic Activity Assay

To determine the effect of the CM on the viability of cells, the MTT cell metabolic activity assay (Sigma-Aldrich) was conducted as described earlier [29]. As some of the bacteria from different regions of GI belong to the same species, only selected bacteria were used in the MTT assays. The cell lines were grown in 96-well plates until 70% confluency was reached. Cells were then treated with 100 µL of bacterial conditioned media and mixed with media (RPMI-1640 complemented with 10% fetal bovine serum (FBS), 1% Minimum Essential Media Non-essential amino acid, 1% l-glutamine, and 1% penicillin streptomycin antibiotic), for 24 h at 95% humidity and 37 °C. For negative controls, cells were incubated alone in the absence of bacterial conditioned media (CM). For positive controls, cells were incubated with 0.2% Triton X-100 at 37 °C for 30 min, resulting in the rupturing of cells and 100% cell death. Ten µL of 5 mg of MTT powder dissolved in 1 mL of 1X phosphate buffered saline (PBS) was added to each well, and incubated at 37 °C for 2 h in the dark. Post incubation, the media in each well was discarded without disturbing the cell monolayer and 100 µL of 100% dimethyl sulfoxide (DMSO) was then added to each well and mixed by vigorous pipetting. The plate was kept for 15 min in the dark at 37 °C, and the absorbance was measured via a spectrophotometer at a wavelength of 540 nm. For the blank, a well comprising DMSO only was included, and percentage cell viability/cell metabolic activity was calculated as depicted:

% Cell viability = [(Absorbance _sample_ − Absorbance _blank_)/(Absorbance _negative control_ − Absorbance _blank_)] × 100. The results were subtracted from 100 to obtain % cell viability inhibition.

### 4.7. Cellular Survival Assay

Cell survival assays were performed to evaluate the effects of the bacterial CM on cancer cells as detailed earlier [30]. Cancer cells were grown in 96-well plates until 95% confluency was reached. The cells were then treated with bacterial CM and RPMI-1640 for 24 h at 37 °C at 95% humidity. Negative control cells were treated with CM prepared using non-pathogenic *E. coli* K-12. As a positive control, cells were incubated for 30 min with 0.2% Triton X-100 and kept at 37 °C. The cells were treated with 2.5% Trypsin solution (Invitrogen), and centrifuged at 2500× *g* for 5 min and seeded onto new plates containing growth medium for 24 h at 37 °C with 95% humidity.

### 4.8. Liquid Chromatography-Mass Spectrometry (LC-MS) Analysis

To determine identity of the molecules in the CM from the selected gut bacteria of crocodile, LC-MS analysis was employed as discussed earlier [31]. The molecules were extracted from the CM using chloroform as an extraction solvent in a ratio of 1:3 of chloroform to conditioned media. Next, the metabolite suspension was dried under reduced pressure utilizing a rotatory evaporator. The metabolites were then re-suspended 1:1 ratio of methanol to water. Analysis of samples was completed with the Agilent 1290 Infinity liquid chromatography (LC) system equipped with a Zorbax SB-C18 column, 100 × 2.1 mm i.d., 3.5 μm particle size, coupled to an Agilent 6520 Accurate-Mass Q-TOF mass spectrometer with dual electron-spray ionizing (ESI source) as described previously (Righi et al., 2016). Chromatographic runs were carried out with a gradient of acetonitrile with the solvent flow rate of 0.3 mL per min at 25 °C, and the injector volume of 2 μL. The chromatograms generated from mass spectrometry were used to establish the identity of molecules from the Metlin_AM_PCDL-N-170502.cdb, Metabolite and Chemical Entity Database. Scifinder software was used to identify biological activity of the molecules and their novelty.

### 4.9. Statistical Assessment

The results are indicative of the mean ± standard error of various independent experiments accomplished in duplicates. Differences of statistical significance were evaluated via a 2-sample *t*-test; two-tailed distribution, contrasting the mean of two different experiments carried out in similar conditions. *p* values < 0.05 were utilized for analysis.

## Figures and Tables

**Table 1:** Bacteria isolated from different locations of the gastrointestinal tract of *C. porosus*. Bacteria were isolated from the gastrointestinal tract of *C. porosus* and based on the color, shape, texture and size, different bacteria colonies were cultured separately until pure culture were obtained, which were eventually subjected to 16S rDNA sequencing for identification.

GI Location	Bacteria	Gram Staining	Phylum
Mouth and esophagus	*Atlantibacter hermannii*	Gram-negative	*Proteobacteria*
	*Bacillus aryabhattai*	Gram-positive	*Firmicutes*
	*Brevibacterium sediminis*	Gram-positive	*Actinobacteria*
	*Chryseobacterium yeoncheonense*	Gram-negative	*Bacteroidetes*
	*Deinococcus grandis*	Gram-positive	*Deinococcus-Thermus*
	*Diaphorobacter polyhydroxybutyrativorans*	Gram-negative	*Proteobacteria*
	*Edwardsiella tarda*	Gram-negative	*Proteobacteria*
	*Enterobacter cloacae*	Gram-negative	*Proteobacteria*
	*Microbacterium paraoxydans*	Gram-positive	*Actinobacteria*
	*Ochrobactrum intermedium*	Gram-negative	*Proteobacteria*
	*Pseudomonas aeruginosa*	Gram-negative	*Proteobacteria*
	*Staphylococcus aureus*	Gram-positive	*Firmicutes*
	*Staphylococcus pasteuri*	Gram-positive	*Firmicutes*
	*Tsukamurella spumae*	Gram-positive	*Actinobacteria*
Stomach	*Aeromonas salmonicida*	Gram-negative	*Proteobacteria*
	*Hafnia paralvei*	Gram-negative	*Proteobacteria*
	*Proteus hauseri*	Gram-negative	*Proteobacteria*
	*Pseudomonas aeruginosa*	Gram-negative	*Proteobacteria*
	*Staphylococcus pasteuri*	Gram-positive	*Firmicutes*
Small intestine	*Enterobacter cloacae*	Gram-negative	*Proteobacteria*
	*Enterobacter tabaci*	Gram-negative	*Proteobacteria*
	*Klebsiella pneumoniae subsp. rhinoscleromatis*	Gram-negative	*Proteobacteria*
	*Klebsiella quasipneumoniae subsp. similipneumoniae*	Gram-negative	*Proteobacteria*
	*Ochrobactrum oryzae*	Gram-negative	*Proteobacteria*
	*Proteus hauseri*	Gram-negative	*Proteobacteria*
	*Proteus vulgaris*	Gram-negative	*Proteobacteria*
	*Providencia alcalifaciens*	Gram-negative	*Proteobacteria*
	*Providencia rettgeri*	Gram-negative	*Proteobacteria*
	*Pseudomonas aeruginosa*	Gram-negative	*Proteobacteria*
	*Pseudomonas guezennei*	Gram-negative	*Proteobacteria*
	*Staphylococcus pasteuri*	Gram-positive	*Firmicutes*
Large intestine	*Aeromonas dhakensis*	Gram-negative	*Proteobacteria*
	*Plesiomonas shigelloides*	Gram-negative	*Proteobacteria*
	*Proteus hauseri*	Gram-negative	*Proteobacteria*
	*Proteus mirabilis*	Gram-negative	*Proteobacteria*
	*Proteus vulgaris*	Gram-negative	*Proteobacteria*
	*Providencia alcalifaciens*	Gram-negative	*Proteobacteria*
	*Pseudomonas aeruginosa*	Gram-negative	*Proteobacteria*
Anus	*Aeromonas dhakensis*	Gram-negative	*Proteobacteria*
	*Proteus columbae*	Gram-negative	*Proteobacteria*
	*Proteus mirabilis*	Gram-negative	*Proteobacteria*
	*Proteus terrae*	Gram-negative	*Proteobacteria*
	*Providencia rettgeri*	Gram-negative	*Proteobacteria*

**Table 2:** MTT assay revealed that CM from selected gut bacteria of *C. porosus* affected cell viability.

*Crocodylus porosus* Organ	Bacteria	% Cell Viability Inhibition Using MTT Assay			
		HeLa	MCF-7	PC3	HaCat
Mouth	*P. aeruginosa*	89.5 ± 2.7	64.0 ± 0.4	67.6 ± 3.2	59.0 ± 1.5
Stomach	*P. aeruginosa*	66.6 ± 7.0	51.7 ± 3.0	53.9 ± 6.7	52.4 ± 1.3
	*P. hauseri*	68.7 ± 3.0	45.5 ± 5.6	61.3 ± 6.0	54.2 ± 1.7
	*H. paralvei*	74.2 ± 8.3	50.1 ± 1.0	65.5 ± 5.9	57.8 ± 1.4
	*S. pasteuri*	71.9 ± 8.9	56.7 ± 1.7	67.7 ± 5.9	59.2 ± 1.0
Duodenum	*K. pneumonia*	78.2 ± 2.1	54.5 ± 2.3	70.8 ± 0.9	55.7 ± 0.4
	*P. guezennei*	52.0 ± 2.8	39.6 ± 1.4	51.3 ± 2.2	44.8 ± 2.9
	*O. oryzae*	66.3 ± 4.4	55.9 ± 6.2	56.3 ± 7.1	50.1 ± 3.0
Ileum	*P. vulgaris*	62.5 ± 6.7	41.9 ± 2.3	61.3 ± 2.0	57.9 ± 2.5
	*P. rettgeri*	72.2 ± 9.4	51.8 ± 2.0	72.0 ± 7.6	57.1 ± 0.2
	*P. hauseri*	76.3 ± 8.6	50.4 ± 0.4	79.1 ± 7.0	63.2 ± 1.2
	*P. aeruginosa*	52.8 ± 1.8	25.8 ± 4.1	40.2 ± 0.4	45.3 ± 1.4
Large intestine	*A. dhakensis*	44.3 ± 6.4	35.4 ± 5.8	57.8 ± 5.2	58.2 ± 2.1
	*P. aeruginosa*	66.2 ± 5.2	56.6 ± 1.5	46.8 ± 2.1	51.5 ± 0.2
Anus	*P. rettgeri*	66.8 ± 4.4	52.3 ± 3.3	57.8 ± 3.0	49.8 ± 2.0
	*P. columbae*	68.9 ± 2.4	47.0 ± 2.6	63.4 ± 1.0	59.7 ± 3.0

## Data Availability

Not applicable.

## Supplementary Materials

The following are available online, Table S1: MTT assay revealed that CM from selected gut bacteria of *C. porosus* did not affect HeLa cell viability. Table S2A: Molecules that were identified from the conditioned media prepared from *P. guezennei* isolated from the duodenum of *C. porosus* through LC-MS. Table S2B: Molecules that remained unidentified from the conditioned media prepared from *P. guezennei* isolated from the duodenum of *C. porosus* through LC-MS. Table S3A: Molecules that were identified from the conditioned media prepared from *P. aeruginosa* isolated from the small intestine of *C. porosus* through LC-MS. Table S3B: Molecules that remained unidentified from the conditioned media prepared from *P. aeruginosa* isolated from the small intestine of *C. porosus* through LC-MS. Table S4A: Molecules that were identified from the conditioned media prepared from *A. dhakensis* isolated from large intestine of *C. porosus* through LC-MS. Table S4B: Molecules that remained unidentified from the conditioned media prepared from *A. dhakensis* isolated from large intestine of *C. porosus* through LC-MS.
