# Supplementary material for: Crocodylus porosus Gut Bacteria: A Possible Source of Novel Metabolites
Source: Molecules. 2021 Aug 18;26(16):4999. doi: 10.3390/molecules26164999 (PMC8398445; doi:10.3390/molecules26164999)
Supplement: Supplementary file 1 [file molecules-26-04999-s001.zip › molecules-1310648-supplementary.pdf]

Table S1: MTT assay revealed that CM from selected gut bacteria of *C. porosus* did not affect HeLa cell viability.

***Crocodylus porosus* organ**	**Bacteria**	**% cell viability inhibition using MTT assay**
		**HeLa**
Mouth & Oesophagus	*Atlantibacter hermannii*	4.5
	Bacillus aryabhattai	3
	Brevibacterium sediminis	6.4
	Chryseobacterium yeoncheonense	2.2
	Deinococcus grandis	4.7
	Diaphorobacter polyhydroxybutyrativorans	1.7
	Edwardsiella tarda	7.8
	Enterobacter cloacae	9
	Microbacterium paraoxydans	7.6
	Ochrobactrum intermedium	4
	Staphylococcus aureus	9.8
	Staphylococcus pasteuri	7.8
	Tsukamurella spumae	5.4
Stomach	*Aeromonas salmonicida*	8.6
Small intestine	*Enterobacter cloacae*	7
	Enterobacter tabaci	8.2
	Klebsiella pneumoniae subsp. Rhinoscleromatis	7.6
	Klebsiella quasipneumoniae subsp. Similipneumoniae	9.2
	Ochrobactrum oryzae	3.2
	Providencia alcalifaciens	5
	Pseudomonas guezennei	9.5
	Staphylococcus pasteuri	8.3
Large intestine	*Plesiomonas shigelloides*	4
	Proteus hauseri	3.3
	Proteus mirabilis	5.4
	Proteus vulgaris	2
	Providencia alcalifaciens	4.4
Anus	*Aeromonas dhakensis*	9.8
	Proteus mirabilis	6.4
	Proteus terrae	5.5

Table S2A: Molecules that were identified from the conditioned media prepared from *P. guezennei* isolated from the duodenum of *C. porosus* through LC-MS. The conditioned media prepared from *P. guezennei* was subjected to chloroform extraction and the extracts were subject to LC-MS analysis. The spectre generated were searched in the METLIN library in order to reveal the potential identity of the detected molecules.

	Compound	Mass	*m/z*
1	**L-Galactose**<br>Molecular formula: $C_6H_{12}O_6$<br>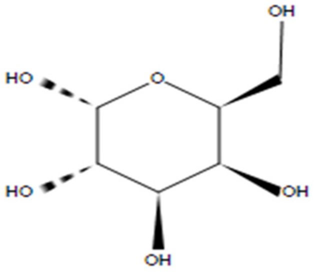 	180.06	215.03
2	**Threonylglycine**<br>Molecular formula: $C_6H_{12}N_2O_4$<br>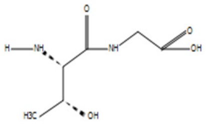	176.08	175.07
4	**Lactic acid**<br>Molecular formula: $C_3H_6O_3$<br>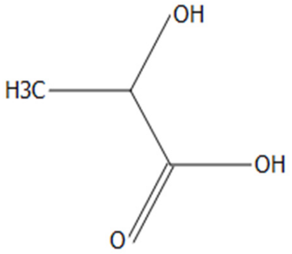 	90.03	125.00
5	**F-Honaucin A**<br>Molecular formula: $C_8H_9FO_4$<br>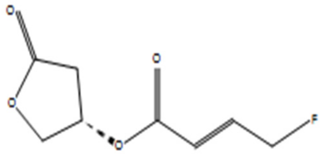	188.05	187.05
6	**D-α-Hydroxyglutaric acid**<br>Molecular formula: $C_5H_8O_5$	148.04	147.03

	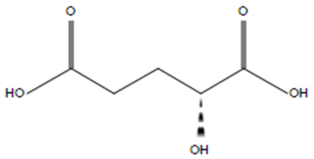		
7	**N-Acryloylglycine**<br>Molecular formula: $C_5H_7NO_3$<br>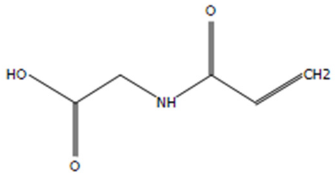	129.04	128.04
8	**Xanthine**<br>Molecular formula: $C_5H_4N_4O_2$<br>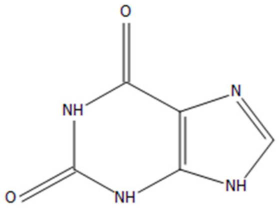	152.03	151.03
9	**(R)-2,3-Dihydroxy-isovalerate**<br>Molecular formula: $C_5H_{10}O_4$<br>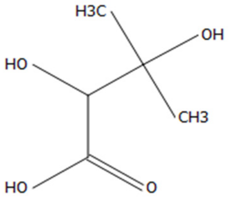	134.06	133.05
10	**Aminocaproic acid**<br>Molecular formula: $C_6H_{13}NO_2$<br>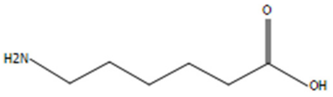	131.09	130.09
11	**Ethyl 3-hydroxybutyrate**<br>Molecular formula: $C_6H_{12}O_3$<br>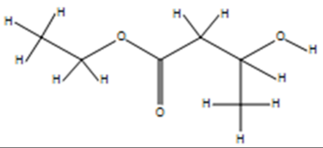	132.08	131.07

12	**3-Indoleacetic Acid**<br>Molecular formula: $C_{10}H_9NO_2$<br>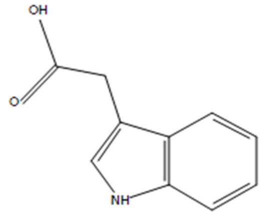	175.06	174.06
13	**2-Caffeoylisocitrate**<br>Molecular formula: $C_{15}H_{14}O_{10}$<br>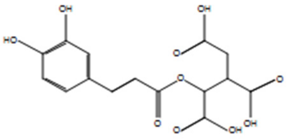	354.06	353.05
14	**3-hydroxy-decanoic acid**<br>Molecular formula: $C_{10}H_{20}O_3$<br>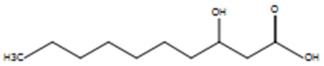	188.14	187.13
15	**2-chloropalmitaldehyde**<br>Molecular formula: $C_{16}H_{31}ClO$<br>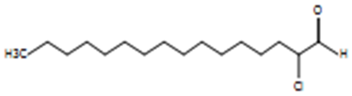	274.21	309.18
16	**Betazole**<br>Molecular formula: $C_5H_9N_3$<br>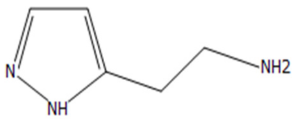	111.08	112.09
17	**Fomepizole**<br>Molecular formula: $C_4H_6N_2$<br>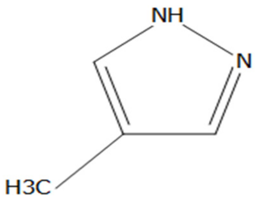	82.05	105.04

18	**2,4-Diamino-6-hydroxylaminotoluene**<br>Molecular formula: $C_5H_5N_5$<br>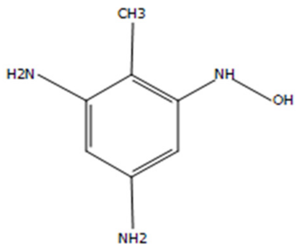	153.09	154.10
19	**Norepinephrine (noradrenaline)**<br>Molecular formula: $C_8H_{11}NO_3$<br>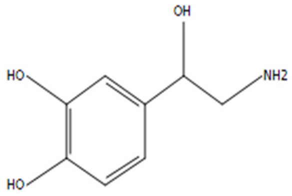	169.07	170.08
20	**Asparagusic acid**<br>Molecular formula: $C_4H_6O_2S_2$<br>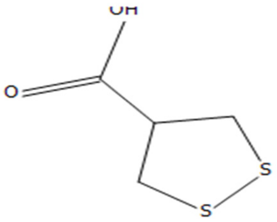	149.98	168.01
21	**N-(1-Deoxy-1-fructosyl)leucine**<br>Molecular formula: $C_{12}H_{23}NO_7$<br>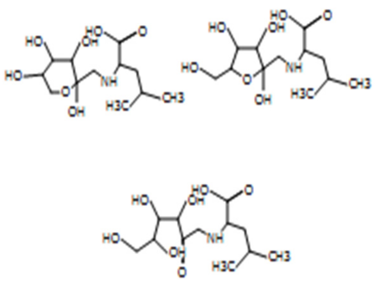	293.15	294.15
22	**3-Nitrosothiazolidine**<br>Molecular formula: $C_3H_6N_2OS$	118.02	119.03

	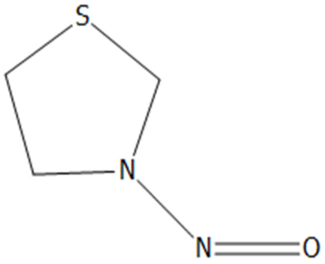		
24	**L-alpha-Amino-1H-pyrrole-1-hexanoic acid**<br>Molecular formula:<br>$C_{10}H_{16}N_2O_2$<br>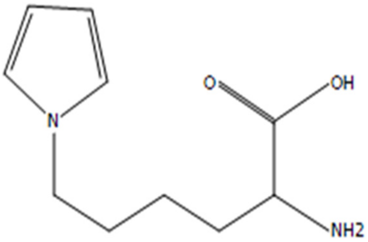	196.12	197.13
25	**Homocysteine thiolactone**<br>Molecular formula:<br>C_4H_7NOS<br>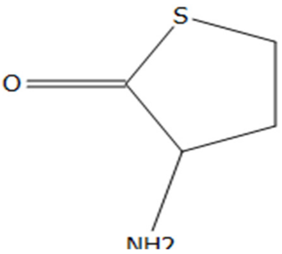	117.03	118.03
26	**L,L-Cyclo(leucylprolyl)**<br>Molecular formula:<br>$C_{11}H_{18}N_2O_2$<br>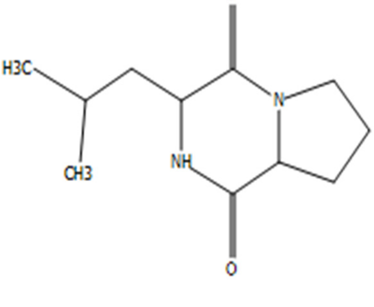	210.14	211.14
27	**Pseudomonine**<br>Molecular formula:$C_{16}H_{18}N_4O_4$	330.13	331.14

	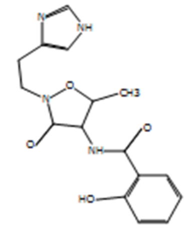		
28	**Kynuramine**<br>Molecular formula:<br>$C_9H_{12}N_2O$<br>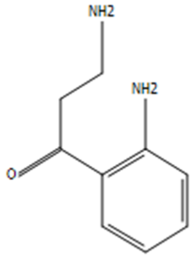	164.09	165.1
29	**12-oxo-10Z-octadecenoic acid**<br>Molecular formula:<br>$C_{18}H_{32}O_3$<br>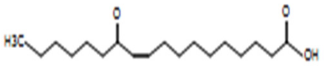	296.24	297.24
30	**9Z,12Z,15E-octadecatrienoic acid**<br>Molecular formula:<br>$C_{18}H_{30}O_2$<br>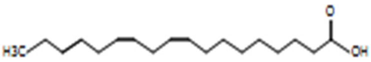	278.22	279.23
31	**Arachidoyl glycine**<br>Molecular formula:<br>$C_{22}H_{43}NO_3$<br>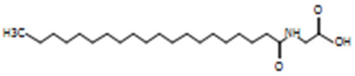	369.32	370.33

Table S2B: Molecules that remained unidentified from the conditioned media prepared from *P. guezennei* isolated from the duodenum of *C. porosus* through LC-MS. The conditioned media prepared from *P. guezennei* was subjected to chloroform extraction and the extracts were subject to LC-MS analysis. The spectre generated were searched in the METLIN library in order to reveal the potential identity of the detected molecules.

Compound	Retention time	Molecular mass	Molecular formula
1	0.649	271.8422	C6 H2 Cl2 S4

2	0.654	213.8924	C4 H Cl3 N2 S
3	0.655	215.8914	C7 H Cl S3
4	0.794	147.9772	C3 H4 N2 O S2
5	0.866	202.0463	C4 H6 N6 O4
6	1.093	118.0291	C5 H2 N4
7	13.579	314.2474	C19 H30 N4
8	15.337	298.1614	C16 H26 O3 S
9	15.498	272.2366	C16 H32 O3
10	15.634	298.1616	C16 H26 O3 S
11	16.328	312.1779	C17 H28 O3 S
12	16.73	312.1777	C17 H28 O3 S
13	17.016	354.2096	C17 H30 N4 O2 S
14	17.345	326.1935	C14 H30 O8
15	17.564	294.1885	C15 H26 N4 S
16	0.657	119.9588	
17	1.002	175.0813	C3 H9 N7 O2
18	1.038	131.0844	
19	1.048	137.0719	C5 H7 N5
20	1.169	105.9904	C3 H6 S2
21	1.698	102.0687	C5 H10 O2
22	8.975	195.0864	C6 H9 N7 O
23	9.918	197	C5 H11 N O S3
24	9.926	229.0873	C11 H16 Cl N O2
25	12.336	157.1466	C9 H19 N O
26	16.678	148.0159	C8 H4 O3
27	20.525	627.1859	C37 H30 Cl N5 O S
28	21.343	701.2069	C38 H43 N3 S5
29	23.941	662.4465	C44 H58 N2 O3

Table S3A: Molecules that were identified from the conditioned media prepared from *P. aeruginosa* isolated from the small intestine of *C. porosus* through LC-MS. The conditioned media prepared from *P. aeruginosa* was subjected to chloroform extraction and the extracts were subject to LC-MS analysis. The spectre generated were searched in the METLIN library in order to reveal the potential identity of the detected molecules.

	Compound	Mass	*m/z*
1	**Pitrazepin**<br>Molecular formula: $C_{19}H_{19}N_5$<br>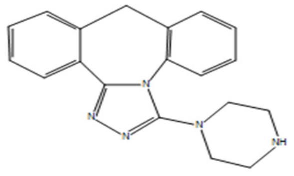	317.16	316.16
2	**3beta-Acetoxy-4-oplopanone**	296.20	331.17

	Molecular formula: $C_{17}H_{28}O_4$ 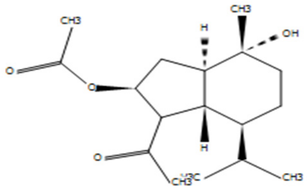		
3	**C11:1n-3** Molecular formula: $C_{11}H_{20}O_2$ 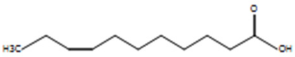	184.15	183.14
4	**O-Acetylcypholophine** Molecular formula: $C_{20}H_{28}N_2O_4$ 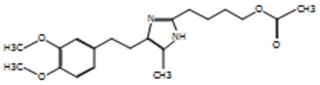	360.21	359.20
5	**cis-9,10-Epoxystearic acid** Molecular formula: $C_{18}H_{34}O_3$ 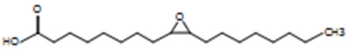	298.25	297.24
6	**3-O-Mycarosylerythronolide B** Molecular formula: $C_{28}H_{50}O_{10}$ 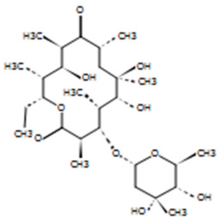	546.34	545.34
7	**(17Z)-1α,25-dihydroxy-26,27-dimethyl-17,20,22,22,23,23-hexadehydrovitamin D3 / (17Z)-1α,25-dihydroxy-26,27-dimethyl-17,20,22,22,23,23-hexadehydrocholecalciferol**	438.31	473.28

	Molecular formula:<br>$C_{29}H_{42}O_3$<br>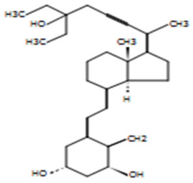		
8	**Thiodiglycol**<br>Molecular formula:<br>$C_4H_{10}O_2S$<br>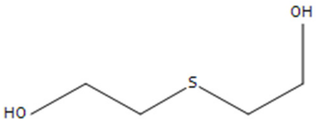	122.04	123.05
9	**1-(1-Pyrrolidinyl)-2-propanone**<br>Molecular formula:<br>$C_7H_{13}NO$<br>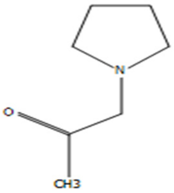	127.10	128.11
10	**Phenylethylamine**<br>Molecular formula:<br>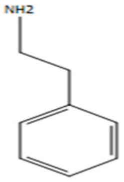	121.09	122.10
11	**2-Methylpropyl formate**<br>Molecular formula:<br>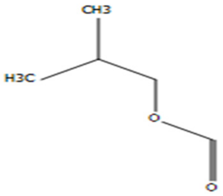	102.07	103.08
12	**(2R,4R)-2-phenylthiazolidine-4-carboxylic acid**<br>Molecular formula:<br>$C_{10}H_{11}NO_2S$	209.05	210.06

	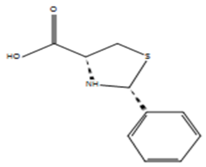		
13	**4-Hydroxypropranolol** Molecular formula: $C_{16}H_{21}NO_3$ 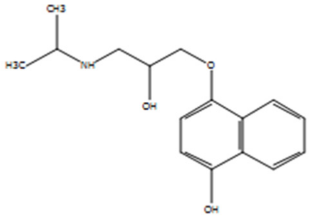	275.15	276.16
14	**2-Hydroxyethylclavam** Molecular formula: $C_7H_{11}NO_3$ 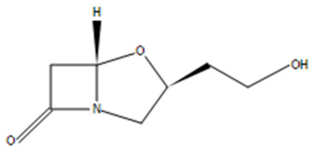	157.07	158.08
15	**N-Methylcalystegine B2** Molecular formula: $C_8H_{15}NO_4$ 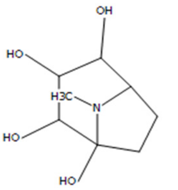	189.10	190.11
16	**9Z,12Z,15E-octadecatrienoic acid** Molecular formula: $C_{18}H_{30}O_2$ 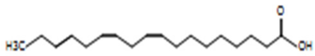	278.22	279.23
17	**5-(1-oxopropan-2-yl)isolongifol-5-ene** Molecular formula: $C_{18}H_{28}O$	260.21	261.22

	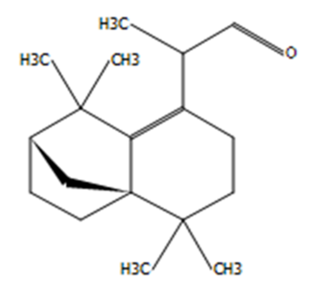		
18	**12-oxo-9E-octadecenoic acid**<br>Molecular formula: $C_{18}H_{32}O_3$<br>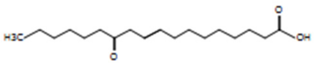	296.24	319.22
19	**11-cis-Dehydroretinal**<br>Molecular formula: $C_{20}H_{26}O$<br>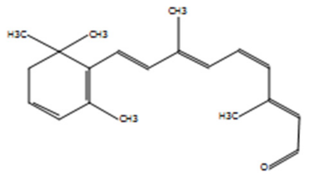	282.20	300.23
20	**Acetaldehyde hexyl isoamyl acetal**<br>Molecular formula: $C_{13}H_{28}O_2$<br>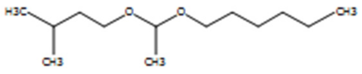	216.21	217.22
21	**Bicyclomahanimbine**<br>Molecular formula: $C_{23}H_{25}NO$<br>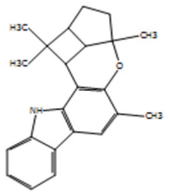	331.19	332.20
22	**N-(1-Deoxy-1-fructosyl)valine**<br>Molecular formula: $C_{11}H_{21}NO_7$	279.13	297.16

	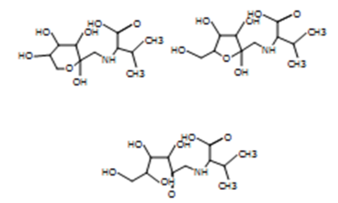		
23	**9,13-dihydroxy-12-ethoxy-10-octadecenoic acid** Molecular formula: $C_{20}H_{38}O_5$ 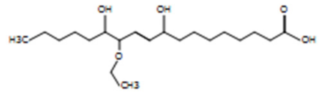	358.27	359.28
24	**3-hydroxy-decanoic acid** Molecular formula: $C_{10}H_{20}O_3$ 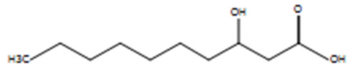	188.14	189.15
25	**Arachidoyl glycine** Molecular formula: $C_{22}H_{43}NO_3$ 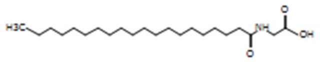	369.34	370.33
26	**Notoginsenoside R10** Molecular formula: $C_{30}H_{50}O_9$ 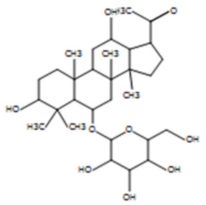	554.34	555.35

Table S3B: Molecules that remained unidentified from the conditioned media prepared from *P. aeruginosa* isolated from the small intestine of *C. porosus* through LC-MS. The conditioned media prepared from *P. aeruginosa* was subjected to chloroform extraction and the extracts were subject to LC-MS analysis. The spectre generated were searched in the METLIN library in order to reveal the potential identity of the detected molecules.

Compound	Retention time	Molecular mass	Molecular formula
1	0.669	147.9732	
2	11.9	266.0004	C6 H6 N2 O10
3	12.588	243.1636	C16 H21 N O

4	13.765	269.1795	C18 H23 N O
5	15.201	476.3002	C25 H40 N4 O5
6	15.354	298.1616	C16 H26 O3 S
7	15.581	549.3533	C28 H47 N5 O6
8	15.628	298.1617	C16 H26 O3 S
9	16.715	312.1776	C17 H28 O3 S
10	16.797	1030.6403	
11	16.799	504.3318	C27 H44 N4 O5
12	16.799	567.3278	C27 H45 N5 O8
13	16.803	589.309	C25 H39 N11 O6
14	17.34	326.1938	C14 H30 O8
15	17.748	530.3468	C29 H46 N4 O5
16	17.75	593.3429	C29 H47 N5 O8
17	17.942	530.2915	C32 H34 N8
18	18.478	532.3627	C29 H48 N4 O5
19	18.479	595.3578	C29 H49 N5 O8
20	19.477	474.2928	C20 H34 N12 O2
21	19.807	845.9589	
22	20.1	356.1695	C16 H28 N4 O S2
23	20.1	441.1464	C21 H23 N5 O4 S
24	21.398	384.1987	C21 H28 N4 O S
25	0.55	181.9781	C8 H3 Cl O3
26	0.589	157.9571	
27	0.872	122.0412	C4 H10 O2 S
28	9.921	229.0874	C11 H16 Cl N O2
29	10.596	222.0471	C10 H10 N2 O2 S
30	10.898	217.132	C10 H19 N O4
31	14.23	307.1555	C14 H26 Cl N O4
32	15.198	476.2991	C24 H44 O9
33	15.44	220.0904	C16 H12 O
34	15.577	503.3457	C26 H49 N O8
35	15.72	614.4525	C34 H58 N6 O4
36	15.816	378.2983	C20 H42 O6
37	15.866	334.2726	C18 H38 O5
38	15.88	290.2454	C16 H34 O4
39	16.479	104.0834	C5 H12 O2
40	16.668	148.0159	C8 H4 O3
41	16.686	305.1558	C18 H24 Cl N O
42	16.792	1030.6401	
43	16.797	504.3302	C26 H48 O9
44	16.831	392.3139	C21 H44 O6
45	16.888	348.2888	C20 H44 S2
46	16.912	304.261	C17 H36 O4
47	17.065	313.2047	C20 H27 N O2
48	17.647	314.1675	C23 H22 O
49	17.747	547.3723	C28 H53 N O9
50	17.938	985.5919	
51	18.476	386.3039	C22 H42 O5

52	18.477	549.389	C29 H51 N5 O5
53	19.869	473.3059	C30 H39 N3 O2
54	20.525	627.1857	C35 H37 N3 S4
55	21.34	701.2061	C44 H32 Cl N3 O4
56	23.96	662.4483	C34 H54 N12 O2

Table S4A: Molecules that were identified from the conditioned media prepared from *A. dhakensis* isolated from large intestine of *C. porosus* through LC-MS. The conditioned media prepared from *A. dhakensis* was subjected to chloroform extraction and the extracts were subject to LC-MS analysis. The spectre generated were searched in the METLIN library in order to reveal the potential identity of the detected molecules.

	Compound	Mass	*m/z*
1	**N-Acryloylglycine**<br>Molecular formula: $C_5H_7NO_3$<br>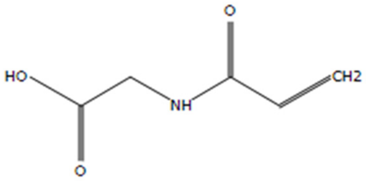 	129.04	1280.4
2	**Erythrono-1,4-lactone**<br>Molecular formula: $C_4H_6O_4$<br>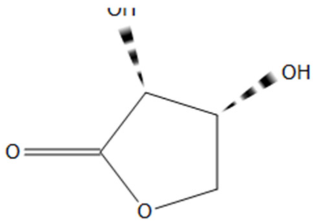 	118.03	117.02
3	**1-(1-Pyrrolidinyl)-2-propanone**<br>Molecular formula: $C_7H_{13}NO$<br>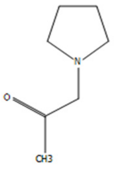	127.10	128.11
4	**5-Methyldeoxycytidine**<br>Molecular formula: $C_{10}H_{15}N_3O_4$<br>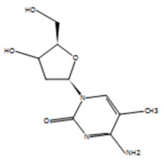	241.11	242.11

5	**His Asn**<br>Molecular formula:<br>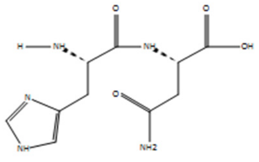	269.11	270.12
6	**2,6-Nonadien-1-ol**<br>Molecular formula: $C_9H_{16}O$<br>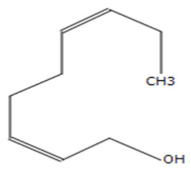	140.12	158.15
7	**2-hydroxy-3-methylhexadecanoic acid**<br>Molecular formula: $C_{17}H_{34}O_3$<br>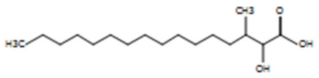	286.25	309.24

Table S4B: Molecules that remained unidentified from the conditioned media prepared from *A. dhakensis* isolated from large intestine of *C. porosus* through LC-MS. The conditioned media prepared from *A. dhakensis* was subjected to chloroform extraction and the extracts were subject to LC-MS analysis. The spectre generated were searched in the METLIN library in order to reveal the potential identity of the detected molecules.

Compound	Retention time	Molecular mass	Molecular formula
1	0.692	147.9733	
2	15.499	272.237	C16 H32 O3
3	16.352	286.2518	C17 H34 O3
4	17.953	530.2909	C32 H34 N8
5	20.104	356.1696	C16 H28 N4 O S2
6	1.023	131.0938	C6 H13 N O2
7	8.93	195.0843	C5 H13 N3 O5
8	9.923	229.0874	C11 H16 Cl N O2
9	16.65	296.157	C23 H20
10	17.945	985.5919	
11	17.948	990.5471	
12	21.108	390.2912	C28 H38 O
13	21.2	370.3143	C20 H42 N4 S
